# Co-occurrence Patterns along a Regional Aridity Gradient of the Subtropical Andes Do Not Support Stress Gradient Hypotheses

**DOI:** 10.1371/journal.pone.0058518

**Published:** 2013-03-07

**Authors:** Ramiro Pablo López, Sergio Valdivia, Mónica L. Rivera, Rodrigo S. Rios

**Affiliations:** 1 Herbario Nacional de Bolivia, Instituto de Ecología (UMSA), Casilla, La Paz, Bolivia; 2 CPEC, Instituto de Ecología, Casilla, La Paz, Bolivia; 3 Laboratorio de Ecofisiología Vegetal – Instituto de Ecología y Biodiversidad (IEB), Departamento de Biología, Universidad de La Serena, Benavente, La Serena, Chile; 4 Laboratorio de Ecología Funcional, Departamento de Biología, Universidad de La Serena, Casilla, La Serena, Chile; Institute of Botany, Czech Academy of Sciences, Czech Republic

## Abstract

The stress gradient hypothesis posits that facilitation and stress are positively correlated. The hump-shaped hypothesis, on the contrary, proposes that facilitation is greater at intermediate stress levels. The relationship between facilitation and environmental stress is commonly studied at small spatial scales and/or considering few species; thus, the implications of facilitation at a community level remain poorly understood. Here, we analyzed local co-occurrence patterns of all plant species at 25 sites within the subtropical Andes to evaluate the role of facilitation and competition as drivers of community structure. We considered a wide latitudinal gradient (19–26°S) that incorporates great variation in aridity. No previous studies have attempted to study these patterns across such a broad scale in warm deserts. Each locality was sampled at two scales (quadrat and patch), and co-occurrence was analyzed via null models. Furthermore, we tested for a relationship between plant co-occurrences and environmental aridity. Resulting patterns depended on life form. When all species were considered, negative associations were found, indicating competition. Woody/cactus life forms tended to be associated across communities, suggesting that there is facilitation between these life forms. Additionally, and unlike previous studies, we found positive associations among shrubs. The strength of the association between woody species changed non-monotonically with aridity. Herbs showed an inverted hump-shaped relationship, albeit ranging mostly among neutral values. Independent of the association type exhibited by different life forms, our community level results do not support current stress gradient hypotheses.

## Introduction

Facilitation has been invoked as one of the main processes driving plant community dynamics in stressful environments [1], [2], [3], as well as in other less arid systems [4], [5]. The main mechanisms underlying facilitation among plants are related to improved microenvironmental conditions and soil properties, as well as protection from herbivores [1]. The main hypothesis addressing facilitative interactions, the stress gradient hypothesis (SGH) [6], postulates that facilitation becomes more frequent as environmental stress increases. While several studies have provided evidence for the SGH (e.g., [7], [8], [9]), others have failed to do so (e.g., [10], [11]). On the one hand, Maestre & Cortina [11], Michalet et al. [12], and Holmgren & Scheffer [13] proposed that the relationship is indeed unimodal (hump-shaped hypothesis), and that facilitation seems to be more important at intermediate stress levels. On the other hand, Liancourt et al. [14] and Gross et al. [15] have postulated that only species far from their ecological optima should be considered as stressed (strained was the exact term they employed, following Welden & Slauson [16]). Based on this argument, Soliveres et al. [17] provided an explanation about the relationship between stress and interaction type, which suggests that the intensity and importance of facilitation at the community level are constant across stress gradients, because the fraction of stressed or strained species should not vary much from one point to another along the gradient, and thus great changes in facilitation along a gradient are not to be expected.

Currently, we know that the variability in the results obtained by previous studies depends on several factors not considered by the SGH, such as species identity [14], the performance under consideration [18], the type of stress (i.e., resource vs. non-resource) [12], [19], and indirect interactions [20], [21]. The impact of a given stress factor can either affect the benefactor effects leading to a collapse in plant interactions or affect the beneficiary responses leading to a shift in plant interactions (see, e.g., [22]). Two important aspects that have influenced the results are related to the breadth of the gradient studied [11], [23]. First, different studies have used different segments of a given gradient, thus obtaining different patterns depending on the part of the gradient considered. Second, the extremes of the gradients are not usually included (but see [9], [24]), although it is at the extremes of gradients where the biggest differences should appear [13], [17].

A very important issue is the one related to the scale of the study. The great majority of studies have considered only interactions between a couple of species or, at most, a few species [19]. Relatively few have focused on the community as a whole (e.g., [25], [26], [27], [28], [29]), although the SGH should be assessed at this scale [19], [23]. Most of these studies have been carried out in alpine regions, where cold is the main stress. There is no empirical evidence from deserts, where much of the theory was developed [3]. Therefore, the response of entire plant communities to aridity gradients remains poorly understood.

In this study, we evaluate how species co-occurrence patterns change across a wide aridity gradient at the community level. The patterns are interpreted in terms of changes in the net outcome of interactions. Although we are aware that other causes, such as environmental heterogeneity or dispersal, are also plausible explanations for positive plant associations, finding a similar pattern of positive associations across several localities distributed along a whole region (arid subtropical Andes) supports a facilitative interpretation rather than alternative ones. The same applies for the case of competition.

Here, we employ an observational approach. Although cause-effect relationships cannot be established unambiguously with observational studies, the spatial scale of the study (whole communities, –i.e., multiple species considered simultaneously–evaluated along a geographic gradient) warrants necessarily this kind of approach [19], [30]. The use of observational approaches has already been highlighted by two influential papers that reconcile contradictory findings in facilitation research [19], [23]. Previous research has employed successfully similar approximations [17], [25], [29], [30], and at least one [25] has demonstrated a close relationship between observational and experimental results. Therefore, observational studies can provide valuable information for understanding whole community dynamics, and are a much needed complement of experimental approaches. In this sense, this study incorporates three important criteria for a better understanding of desert communities: a geographical region, a wide aridity gradient, and a whole-community approach.

Specifically, we searched for evidence that facilitation might be an important process in these arid and semiarid communities of the Andes. Albeit facilitation has been widely documented in deserts, this has rarely been established at a community level. We considered that a recurrent pattern of positive associations between species in the region studied would constitute a good indicator of facilitation as a dominant process. Moreover, we evaluated if positive spatial associations between species tend to increase with aridity. We think that this is a way of evaluating the SGH and other hypotheses related to stress gradients. We have the premise that if facilitation is important for the community, a community-level analysis should be able to detect it.

Our analyses were based on the following hypotheses: 1) shrubs of the arid subtropical Andes facilitate seedlings; thus, if this effect is important at a community level, null models should reveal positive spatial associations across study sites; 2) if facilitation increases with aridity, so will the prevalence of positive associations (according to SGH). Likewise, if facilitation increases with aridity up to a certain aridity level (intermediate aridity conditions) and then decreases (hump-shaped hypothesis), positive associations will exhibit a peak at intermediate aridity levels.

## Methods

### Field Sampling

**Figure 1:**
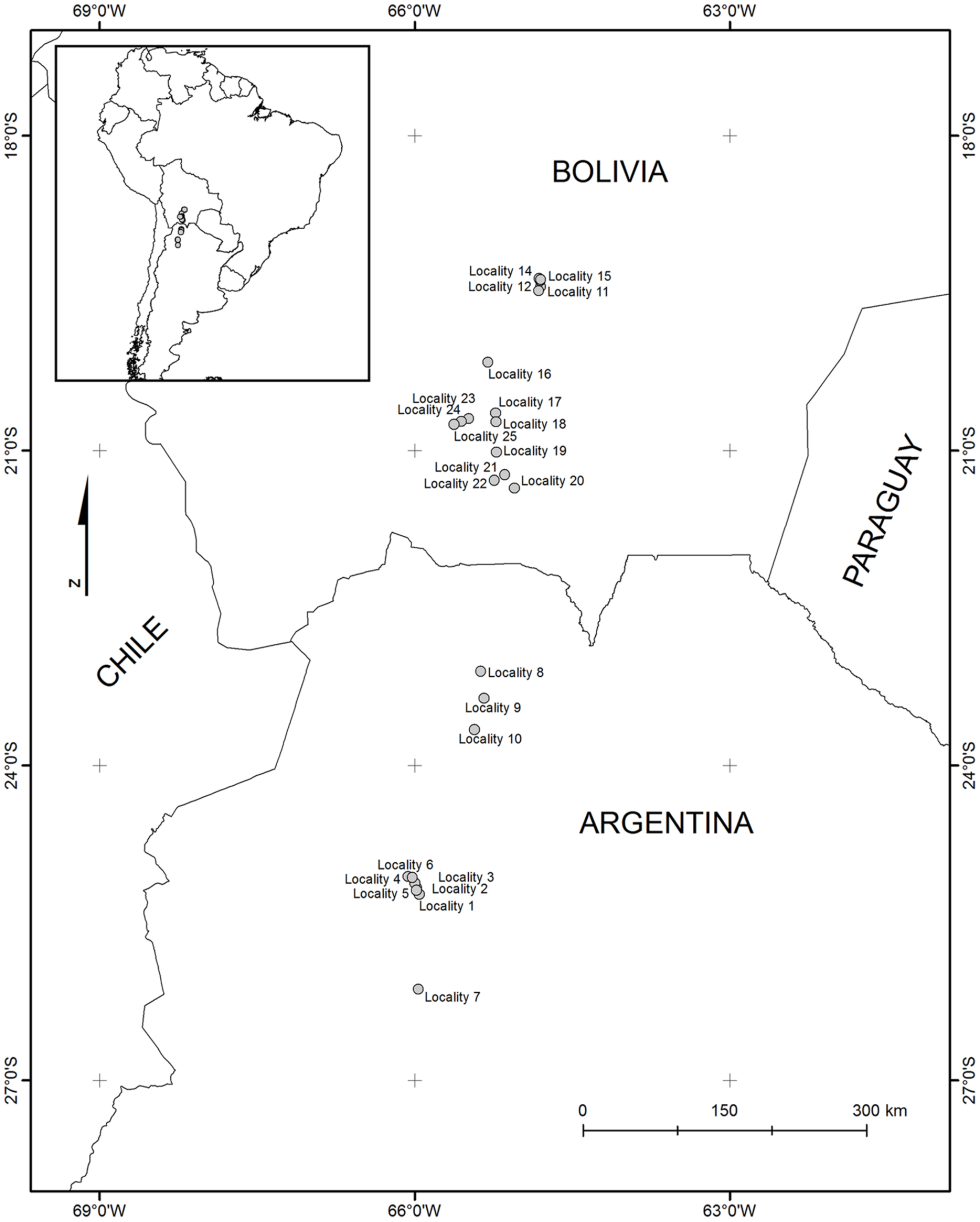
The study region, showing the geographical locations of each of the 25 localities. These are grouped in four subregions, each separated from the other by high altitude (Puna) ecosystems.

Fieldwork took place during the rainy season of 2010 (February–March). Twenty-five localities (sites) in southern Bolivia/northern Argentina were sampled (Figure 1, Table 1). All localities are found in the Andes (19–26° latitude, an almost 800 km-long latitudinal gradient). They fall at an intermediate elevation (2000–3300 m), mostly in the Prepuna region [31], [32], [33]. No specific permissions were required for these activities, except for localities 1 through 6, which were conducted in Los Cardones National Park; for these localities, the required authorization was obtained from the National Parks Administration (APN), Argentina’s authority for the national parks. The locations for which specific permission was not required were not privately-owned or protected in any way. The study did not involve handling or collection of endangered species.

**Table 1:** Geographic, climatic, and vegetation characteristics of the 25 sites studied.

Locality	Latitude/longitude	Altitude(m asl)	Species richness (total number in each site)	Total plant cover (%;includes all life forms)	Shrub cover (%)	Precipitation (mm)	Temperature (°Celsius)	de Martonne aridity index (pp/T°+10)	Dominant woody species
Locality 1	25 13 40.1S 65 57 41.5 W	2946	9	30.1	6.3	90	10.5	4.4	*Larrea divaricata, Flourensia fiebrigii, Baccharis* sp.
Locality 2	25 10 10.4S, 65 59 00.4W	2934	6	26	6	90	11	4.3	*Larrea divaricata, Junellia longidentata*
Locality 3	25 07 26.0S, 66 00 12.0W	2967	8	31.8		100	10.4	4.9	*Larrea divaricata, Salvia* sp.
Locality 4	25 03 42.7S, 66 04 08.9W	2539	12	34.7	15.3	120	12.5	5.3	*Bulnesia schickendantzii, Larrea divaricata, Cercidium praecox*
Locality 5	25 11 23.9S, 65 59 21.1W	2867	5	39	6.1	90	11.2	4.2	*Larrea divaricata, Junellia longidentata, Prosopis ferox*
Locality 6	25 04 13.7S, 66 01 29.7W	2730	7	48.7		120	11.5	5.6	*Prosopis torquata, Larrea divaricata, Opuntia sulphurea, Lycium* aff. *tenuispinosum*
Loality 7	26 08 07.5S, 65 58 07.5W	1664	16	50.5		200	15.9	7.7	*Senna crassiramea, Bulnesia schickendantzii, Junellia longidentata, Lycium* aff. *tenuispinosum*
Locality 8	23 06 19.2S, 65 22 30.2W	3227	7	52.5	31	180	10.3	8.9	*Baccharis boliviensis, Prosopis ferox, Parodia maassii*
Locality 9	23 21 46.2S, 65 20 41.9W	2781	13	39.5	25	120	12.5	5.3	*Senna crassiramea, Airampoa airampo, Parodia maassii, Cercidium andicola, Prosopis ferox*
Locality 10	23 39 28.8S, 65 26 11.1W	2430	10	47.5	26	120	13	5.2	*Senna crassiramea, Lycium tenuispinosum, Cercidium andicola*
Locality 11	19 26 22.9S, 64 48 17.6W	2159	20	48.7		453	20.8	14.7	*Acacia furcatispina, Aspidosperma quebracho-blanco, Jatropha* sp., *Prosopis* sp.
Locality 12	19 28 39.1S, 64 49 17.8W	2030	10	45.2		453	21	14.6	*Senna crassiramea, Aspidosperma quebracho-blanco, Acacia furcatispina*
Locality 13	19 23 06.4S, 64 48 30.1W	2492	17	42.9		453	19.6	15.3	*Prosopis sp., Bougainvillea spinosa, Acacia macracantha*
Locality 14	19 21 32.7S, 64 49 12.4W	2664	14	42.3		453	18.5	15.9	*Baccharis sp., Prosopis sp., Solanum sp., Aspidosperma quebracho-blanco*
Locality 15	19 22 21.4S, 64 48 05.2W	2421	15	39.1		453	19.5	15.4	*Prosopis* sp., *Acacia aroma, Berberis* sp., *Aspidosperma quebracho-blanco*
Locality 16	20 09 33.0S, 65 18 21.6W	3338	8	71.2		350	13	15.2	*Baccharis boliviensis, Prosopis ferox*
Locality 17	20 38 42.8S, 65 13 57.9W	2612	13	51.1		300	18	10.7	*Flourensia fiebrigii, Cercidium andicola, Helogyne straminea, Dodonaea viscosa*
Locality 18	20 43 42.8S, 65 13 46.5W	2426	8	39.6	20.4	320	18	11.4	*Coursetia heterantha*
Locality 19	21 01 07.3S, 65 13 29.5W	2394	7	27.6		320	18.2	11.3	*Cercidium andicola, Senna crassiramea*
Locality 20	21 21 28.7S, 65 03 13.2W	3023	11	36.9	26	250	17.5	9.1	*Prosopis ferox, Opuntia sulphurea, Atriplex* sp., *Lycianthes* sp.
Locality 21	21 13 48.8S, 65 08 52.0W	2638	14	37.4	31	310	19	10.7	*Opuntia sulphurea, Acacia feddeana, Prosopis sp., Lycinathes* sp.
Locality 22	21 16 55.4S, 65 14 39.7W	2486	7	20.2	26	320	18.6	11.2	*Larrea divaricata, Cercidium andicola, Prosopis ferox*
Locality 23	20 41 50.5S, 65 29 20.4W	3009	11	46.1	44.7	350	16.3	13.3	*Acacia feddeana, Croton* sp., *Prosopis ferox, Cercidium andicola, Opuntia sulphurea*
Locality 24	20 43 20.1S, 65 33 38.5W	3106	10	42.9	22.2	350	16.2	13.4	*Prosopis ferox, Opuntia sulphurea*
Locality 25	20 45 04.4S, 65 37 50.3W	3119	21	42.5	42	350	16	13.5	*Prosopis ferox, Opuntia sulphurea, Caesalpinia trichocarpa*

Three separate, although floristically related, regions were sampled. The first one included the most septentrional and, in general, less arid localities (400–500 mm), around 19° latitude (Bolivian dry valleys). The second region was located within the Bolivian Prepuna (20–22° latitude, precipitation: 200–350 mm). The last region (arid localities) encompassed the Argentinian Prepuna and parts of the Monte Desert (23–26° latitude, rainfall: 90–250 mm). Thus, the gradient ranged from semiarid to extremely arid based on the de Martonne aridity index (Table 1). This index is obtained by dividing mean precipitation by mean temperature values plus 10. Plant communities were floristically related (similarities to genus level), and some species were found in all three regions.

In our sampling design two scales were considered: the quadrat scale and the vegetation patch scale. The quadrat scale was aimed mainly at sampling herbaceous species (including their relationship with woody species). At each locality, 100 40×20-cm frequency quadrats were laid out in a systematic manner, i.e., along 10 parallel, 50-m lines traced every 10 m. Along each line, the sampling frames were placed at 5 m intervals. In each quadrat, shoot frequency [34] was determined by recording the presence of all plants having shoot projections inside the quadrat. One advantage of this procedure is that it allows the recording of all plants, regardless of their life form, using a common abundance parameter. Abundance is determined as the proportion of quadrats in which a given species was recorded (i.e., frequency). Plant specimens were collected for identification.

The vegetation patch scale was implemented for bigger life forms (e.g., shrubs and cacti) as follows. The quadrats used for the quadrat scale served for the patch scale as well, as they were used for selecting the patches. Besides the recording of herbaceous species, if a shrub (focal shrub) was recorded in a given quadrat, shrub or cactus species that had their canopies below or above the focal shrub or that had their crowns imbricated with that of the shrub (high crown overlap: when >50% of the radius of at least one of the shrubs was mixed with the other shrub’s canopy) were recorded. We considered all shrubs >50 cm and all cactus species. If two or more shrubs were recorded in the quadrat, the shrub having greater crown projection was considered as the focal individual. In almost all cases shrubs having less cover were also part of the patch thus identified. Yet, other shrub species that were associated with the shrubs with less canopy cover inside the quadrat were not considered, so that the patch did not always include all shrubs forming the patch. In this sense, the patch tended to include all the species that were truly spatially associated and that were very likely interacting strongly. We proceeded this way because quadrat placement was our reference to select shrub associations, and because we did not intend to sample patches as separate entities from open ground (see data analysis). The scales considered are well within the range (≈<1 m–3 m) in which facilitative and competitive interactions take place in dry/arid environments (see, e.g., [35], [36], [37]). Our sampling protocol involved sampling sites with no shrub/cactus species (empty sampling units), as around 20–45% of the sampling positions had no shrubs; these empty sites were included in the analysis (see below).

### Data Analysis

Climatic data for all localities were obtained from the nearest meteorological stations. If the nearest meteorological station happened to be at a different altitude from that of a given study site, precipitation values were kept the same while temperature values were calculated considering a linear lapse rate of −0.6°C/100 m, a value used at regional and continental scales when there is a lack of reliable meteorological data [38]. In this way we considered the influence of temperature on water availability, given that the latter is not a function of precipitation alone.

For each locality species were ordered in the form of a presence-absence matrix (species in rows, quadrats in columns); hence, 25 matrices were analyzed. Species that could not be distinguished in the field (e.g., globose cacti and a few herbaceous species) were each considered as a single species. Matrices were analyzed via null models that consider the checkerboard score (C-score) index, which measures how often different species pairs appear in the same quadrats [39]. This is considered one of the best indexes to determine species co-ocurrence patterns for sample lists [39]. In addition, we calculated the variance ratio (V ratio) index for the same data, but since it was highly correlated with the C-score (r = 0.90 to 0.96, depending on the set of life form), only analyses with the former are presented here. An observed C-score was calculated for each locality and all 25 indices were compared to indices derived from 5000 null matrices (randomly assembled matrices). The null model employed to generate null matrices was based on the fixed-equiprobable algorithm, which has low type I error, good power to detect non-random patterns, and is recommended when the data matrix has many zeros and sampled communities are homogeneous [39]. Fixed refers to the rows, and indicates that the mean of the number of occurrences of each species in the null communities is the same as in the original data set; equiprobable alludes to the columns (quadrats), and indicates that that they are equally likely to be represented in the null communities. Null matrices were created with a sequential swap algorithm by repeatedly swapping randomly chosen submatrices of the form 01/10 [39].

We chose the fixed-rows, equiprobable-columns algorithm instead of the fixed-rows, fixed-columns algorithm because we believed that all the plots within a matrix were equally suitable to all species (hence our systematic sampling). This was based on results of our previous research (see, e.g., [40], [41]) in the region which showed that dominant shrubs recruited in open as well as beneath-shrub spaces. In that sense, our analysis considered the sampled communities as homogeneous. For us, shrub patches and open spaces were variants of the same community. Indeed, shrub patches are not completely discrete (as true islands), something the fixed-fixed model requires, as there is a continuum between open spaces and the center of shrubs (in fact, most species of herbs grow at least in the periphery of the shrubs’ crowns). These characteristics are appropriate for fixed rows, equiprobable columns algorithms [39]. Thus, our sampling involved the existence of degenerate matrices (samples without species records; all our localities had empty sites, especially for the patch scale).

Because raw C-score values vary depending on species number, abundance and co-occurrences observed at each locality, we calculated a standardized effect size (SES) for each matrix in order to be able to compare results across sites (see, e.g., [26], [29], [42], for a similar approach). SES’s were calculated as: (observed C-score - mean of simulated C-scores)/standard deviation of simulated C-scores. Hence, the SES measures the number of standard deviations that the observed index is above or below the mean index of the simulated communities. If values are positive, then there are less co-occurrences than expected by chance, whereas if they are negative, then there are more co-occurrences. The first case is indicative of competition, while more co-occurrences is of facilitation. Assuming a normal distribution of deviations, approximately 95% of the SES values should fall between −2 and 2 if co-occurrences are not different from what is expected by chance alone.

The 25 data matrices obtained for the quadrat scale were analyzed for all species combined and, in a separate analysis, only for herbs. In the latter case, assessments were carried out with the same quadrats used for the all-species analysis, but omitting the records of other life forms. The vegetation patch scale analysis was conducted for woody species (including cacti), but a similar analysis was done excluding cacti (to achieve this, cacti were omitted from the record for the patch and only shrubs and small trees were retained). We considered this latter analysis to be important as we have evidence that facilitation in these Andean environments seems particularly important for cacti (e.g., [40]), and we expected that excluding them from the analysis might bring about changes in the strength of the species association. The SES analysis was performed with EcoSim7 [43].

The SGH was tested by modeling co-occurrence in terms of aridity using regression analysis. We used generalized linear models (GLM) with normal error and an identity-link function to regress the SES from all localities against their respective aridity index values (Table 1). A positive relation would provide evidence for the SGH (i.e., the higher the values of the aridity gradient the more positive the C-score). The same analytical procedure was applied to both the quadrat and vegetation patch scale and for sets of lifeforms separately. In all cases we employed 1^st^, 2^nd^ and 3^rd^ order polynomial regressions in order to identify linear and non-linear relations and assess the best fit to the data. Fitted models were compared using information-theory-based framework for model-selection and the second-order Akaike information criteria (AICc). We ranked all models from best to worst on the basis of the lowest AICc values. To find competing models we calculated ΔAICc values (the difference between the AICc value for a given model and that with the lowest AICc) and then generated a likelihood weight (*w_i_*) for each competing model (competing models have a ΔAICc <2). The *w_i_* for each model, represents a bayesian posterior model probability that from the set of models considered, model *i* would be the “AICc-best” model and thus helps to discriminate among competing models [44]. Significance of individual parameters within the models was based on log likelihood ratio tests.

## Results

**Figure 2:**
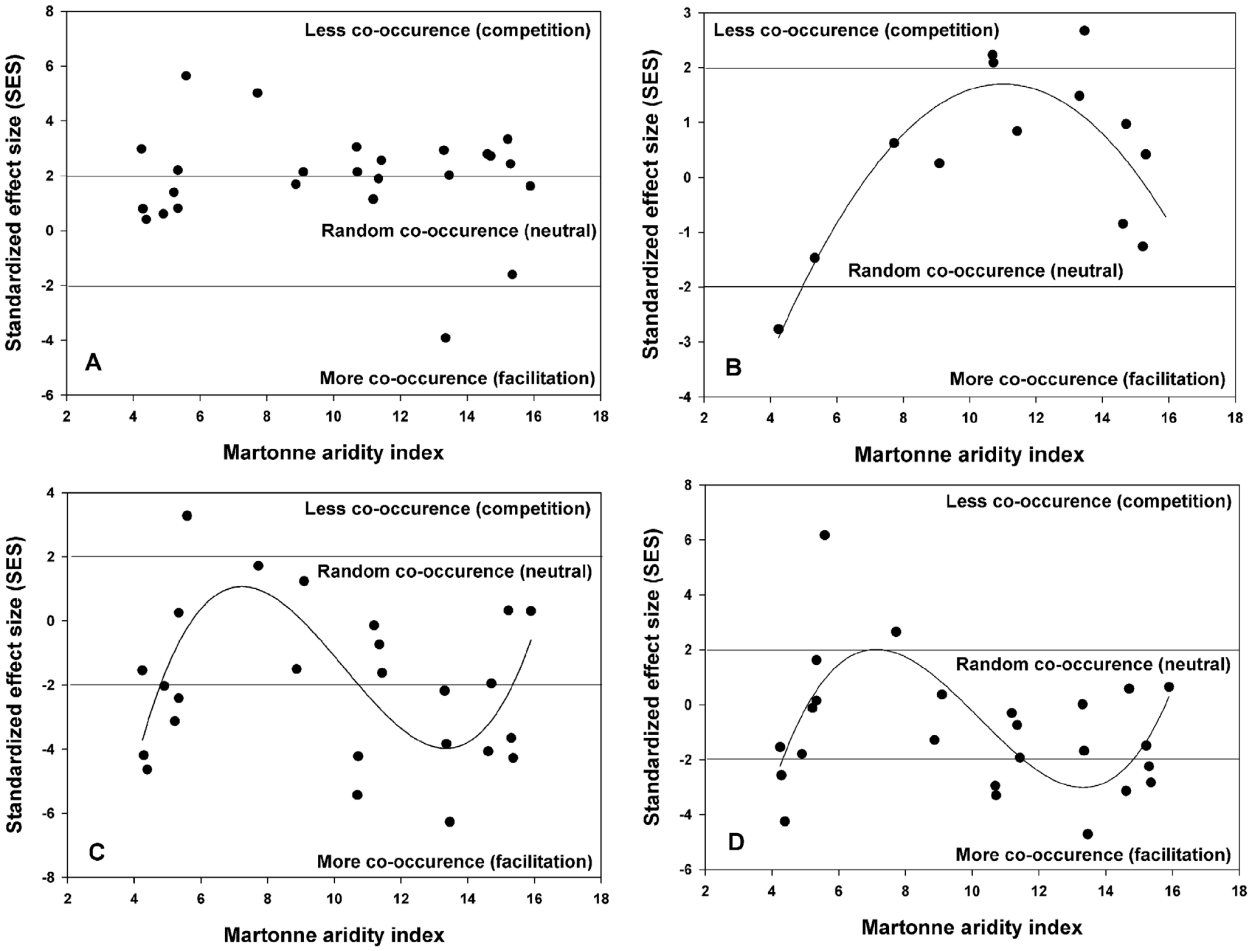
Pattern of association between aridity and species co-occurrence across 25 localities of the arid Andean subtropics. Species within communities tend to co-occur more than expected by chance and nonlinear pattern emerge according to lifeform with the aridity gradient. A) Values for all species sampled within communities; B) values for all herbs sampled within communities; C) values for shrubs and cacti sampled within communities; and D) values for only shrubs sampled within communities. Co-occurrence estimates are based on the standardized effect size (SES) of C-score values. Values of SES lower than −2 are indicative of a predominance of facilitation within communities, whereas values greater than 2 of competition. The null model employed for all 25 localities is based on a fixed-equiprobable algorithm.

When the degree of co-occurrence among all species across communities is considered, most localities exhibit positive SES values (>2) (Table 2), indicating less patterns of association than expected by chance alone. In only one locality (locality 24) was there evidence of strong association. Patterns change, however, when different life forms are evaluated. When herbs are considered, SES values indicate a neutral association among species (Table 2, Fig. 2). Only in localities one and three, respectively, was there evidence for more or less co-occurrence than expected by chance. When shrubs and cacti were considered, the number of localities with negative SES values increased significantly and several had values <−2 (Table 2, Fig. 2). Patterns of shrubs alone (without cacti) resulted in fewer negative SES values, indicating that the association with shrubs is more important for cacti. Nonetheless, some SES values remained significantly negative, indicating that facilitation may also be important for some shrubs.

**Table 2:** Species co-occurrence across localities (a) with different aridity levels (b) within the subtropical Andes; standardized C-score values (SES) for all species combined (c) and for specific life forms (d-f) are reported.

Locality	Aridityindex	Allspecies	All herbs	Shrubs-cacti	Only shrubs
Locality 1	4.4	0.40691		−4.64088	−4.24
Locality 2	4.3	0.79514		−4.19114	−2.57
Locality 3	4.9	0.61093		−2.0322	−1.79
Locality 4	5.3	2.20259	−1.47255	0.24995	0.15
Locality 5	4.2	2.97854	−2.76824	−1.54121	−1.54
Locality 6	5.6	5.64039		3.27761	6.17
Locality 7	7.7	5.0131	0.62111	1.71396	2.65
Locality 8	8.9	1.69009		−1.5057	−1.28
Locality 9	5.3	0.8145		−2.41171	1.62
Locality 10	5.2	1.394		−3.1262	−0.12
Locality 11	14.7	2.71831	0.9704	−1.94852	0.58
Locality 12	14.6	2.79002	−0.84992	−4.06675	−3.14
Locality 13	15.3	2.43462	0.41662	−3.65495	−2.24
Locality 14	15.9	1.62111		0.30538	0.65
Locality 15	15.4	−1.60399		−4.27407	−2.83
Locality 16	15.2	3.32577	−1.26081	0.32552	−1.48
Locality 17	10.7	2.13851	2.09281	−4.22472	−3.30
Locality 18	11.4	2.55447	0.84494	−1.62065	−1.93
Locality 19	11.3	1.8918		−0.73693	−0.74
Locality 20	9.1	2.13268	0.25582	1.23911	0.37
Locality 21	10.7	3.04742	2.23342	−5.43278	−2.95
Locality 22	11.2	1.1443		−0.14161	−0.30
Locality 23	13.3	2.92994	1.48698	−2.18039	0.01
Locality 24	13.4	−3.91524		−3.83374	−1.68
Locality 25	13.5	2.01855	2.6743	−6.26646	−4.71

Negative values indicate positive associations, while positive ones indicate negative associations (values greater than +2 or lower than −2 being considered significant). Empty cells indicate very low herb abundance in the respective sites, which prevented us from conducting co-occurrence analysis based on null models.

When co-occurrence patterns for all-species are related to aridity, there is no trend towards an increase in species association with an increase in aridity, as predicted by the SGH (non-significant 1^st^ order polynomial coefficients, Table 3), nor towards the existence of a hump at intermediate aridity levels (Fig. 2a, non-significant 2^nd^ and 3^rd^ order polynomial coefficients, Table 3). Aridity was, however, related to the degree of co-occurrence among all herbs in a non-linear form (significant 2^nd^ order polynomial model with ΔAICc = 0, and significant parameter coefficients, Table 3) revealing the existence of a hump-shaped relationship for this lifeform, although ranging mostly among neutral SES values (Fig. 2b). A hump-shaped relation indicates the tendency towards more competition at intermediate aridity levels, precisely the opposite pattern predicted by the hump-shaped hypothesis, which predicts facilitation to predominate at mid-range of stress gradients. The 2^nd^ order polynomial model has a strong significant linear component (Aridity coefficient = 2.21) that goes from more co-occurrence SES values to less co-occurrence SES values. This linear trend levels off and goes down again to neutral co-occurrence as aridity decreases even more (Fig. 2b, Table 3).

**Table 3:** Linear and non-linear relationships between SES of C-scores and aridity across communities.

Set of life forms	Model	Slope parameter			Pseudo-*R^2^*	AICc	ΔAICc	K	*w_i_*
		Aridity	(Aridity)^2^	(Aridity)^3^					
All species	1^st^ order	−0.05	–	–	0.01	108.4	0	3	0.71
	2^nd^ order	0.38	–0.02	–	0.03	110.76	2.36	4	0.22
	3^rd^ order	3.54	−0.36	0.01	0.07	112.97	4.57	5	0.07
All herbs	1^st^ order	0.18	–	–	0.17	54.42	10.5	3	0.00
	**2^nd^ order**	**2.21**	−**0.1**	**–**	0.74	43.92	0	4	0.91
	3^rd^ order	−0.01	0.1	0.35	0.75	48.69	4.77	5	0.08
Shrubs and cacti	1^st^ order	−0.09	–	–	0.03	119.98	1.59	3	0.29
	2^nd^ order	0.33	−0.02	–	0.04	122.53	4.14	4	0.08
	**3^rd^ order**	**10.78**	−**1.15**	**0.04**	0.28	118.39	0	5	0.63
Only shrubs	1^st^ order	−0.13	–	–	0.06	117.91	2.15	3	0.24
	2^nd^ order	0.14	−0.01	–	0.06	120.63	4.87	4	0.06
	**3^rd^ order**	**0.04**	−**1.15**	**10.63**	0.32	115.76	0	5	0.70

Relationships are shown for all life form sets considered in the study. For each set, linear (1^st^ order), quadratic (2^nd^ order) and cubic (3^rd^ order) models are shown. All models were fitted using general linear models and significance of their parameters estimates are based on Chi-square likelihood ratio tests. Significant coefficient values are shown in bold.

For shrubs and cactus species, the relationship between co-occurrence patterns and aridity was more complex. The 3^rd^ order polynomial regression model gave the best fit to the data (i.e., ΔAICc = 0) with all regression coefficients significant (Table 3). There is a linear component that reveals strong co-occurrence in the most arid localities (end of the gradient) which become neutral or even with less co-occurrences than expected by chance as we move towards less arid localities. As localities decrease in aridity even more the relationship levels off and localities with neutral and more co-occurrences than random reappear and dominate. Finally, in the less arid localities, neutral associations emerge again (Fig. 2c).

The relationship between co-occurrence patterns of shrubs alone and aridity was similar. In this case, however, localities fluctuated more in around the range of neutral SES values, revealing the importance of cacti in generating stronger patterns with aridity. The 3^rd^ order polynomial regression model also gave the best fit to the data and had all regression coefficients significantly different from zero (Table 3). Here, however, the linear component was lower than when cacti are included with the same trend where the most arid localities become neutral or even with less co-occurrences than expected by chance as we move towards less arid localities. Changes in patterns in this case, however, were stronger (cubic coefficient = 10.63, Table 3). As localities decrease further in aridity, co-occurrence decreases and levels off, increasing again with localities neutral or with more co-occurrences than random reappearing. In the less arid localities, neutral associations tend to dominate (Fig. 2d).

## Discussion

Woody/cactus plants of the majority of our 25 communities showed positive associations. One of the processes that could explain this pattern is facilitation, at least within our assumption of the fixed-equiprobable null model (which assumes that all sites are similar for plant colonization). It is unlikely that a similar patchy distribution of resources that could explain the pattern arises in all 25 localities. In addition, if the pattern was the result of site heterogeneity, all life forms would show aggregated patterns. This suggests that facilitation might be an important process for woody plants in desert communities of the subtropical Andes. Previous studies in the region indicate that facilitation is important mainly for cacti (e.g., [40], [45]), supporting our findings.

Although shrubs alone tend to be less associated than cactus with shrubs, some SES values including only shrubs remained significantly negative, implying that facilitation may also be important for shrub species. This is in line with what is known for other arid systems, especially in North America, where most (if not all) woody species are involved, to a greater or lesser degree, in positive associations [35], [46], [47]. Previous studies [40], [41] have suggested that shrubs of several dominant species may not need a nurse to get established. However, shrubs of other species not considered in these studies might indeed need a nurse. Moreover, those studies [40], [41] were carried out within a single year, and it is known that plant responses may change depending on the year (e.g., [10]). Results of the present study reflect instead a more integrated, long-term response.

The all-species and the herb-species analyses, however, showed a different result from the case with shrubs and cacti: mostly positive SES values were shown for the former and neutral for herbs. Positive SES values are indicative of competition [39] as the possible main driving process for the all-species case. More specifically, positive values could be related to the existence of competition between herbs and woody species but could also mean that different herb assemblages are associated with the two main microhabitats (beneath shrubs/open spaces). The vegetation patch scale analysis rules out competition among woody species and the analysis with only herbs showing neutral SES values indicate that the positive SES are not due to herb competition. Moreover, the all-species analysis may have been influenced by a negative association between shrubs detected at the quadrat scale, as 40×20 cm is a size too small to allow detection of more than 1–2 shrub species, thus indicating species segregation.

The results indicating an absence of competition among herbs or those indicating that herbs are not sorted out into separate communities (beneath shrubs/open spaces) should be interpreted with caution. Many individuals usually found in open or bare areas are able to grow in the outer perimeter of shrub’s crowns, and for the present study these individuals were considered to be associated with the microhabitat beneath shrubs. This caused the two communities to loose distinctiveness. Previous studies [48] have found that both microhabitats have different species abundances and even differences in species composition.

For the positive association observed in shrubs, the specific facilitation mechanism involved should be evaluated: amelioration of microsite conditions or protection from herbivores. Seed trapping (e.g., [49]) could help generate the pattern found, but if facilitation was not an important process for the plant community, shrubs would not tend to maintain positive associations.

When all species are considered, the SGH is not supported by our data at a community-level, nor the hump-shaped alternative. This is largely independent of the null model chosen (fixed-equiprobable or fixed-fixed). Neither linear nor non-linear regressions described adequately our data. These patterns change, however, when individual sets of lifeforms are considered. In all cases (all herbs, shrubs and cacti and only shrubs) nonlinear relations emerge. Quite surprisingly, a 3^rd^ order polynomial regression gave the best fit to the data (i.e., shrubs and cacti and only shrubs).

Failure to support the SGH could be a result of several reasons. The significant polynomial regressions found suggest that the influence of multiple environmental factors along such a wide gradient might have masked the influence of aridity alone. The finding of Le Bagousse-Pinguet et al. [22] show, for example, that a given stress factor acts on the beneficiary, but also on the benefactor, frequently affecting them in different ways. This may generate unpredictable effects/responses of benefactors and beneficiaries, respectively, which cannot be anticipated with the simple predictions of the stress gradient hypotheses, proposed to explain the influence of single stress gradients.

One of the most important factors affecting plant-plant relationships in addition to aridity should be the differences in grazing pressure among regions. Grazing has been invoked as a main factor changing the intensity and direction of plant-plant relationships [50], and the different regions may vary in grazing levels, obscuring abiotically driven influences (e.g., [51]). Grazing (or another stress) could be negatively correlated with aridity, and in that case a pattern similar as the one found in our study is to be expected (see, e.g., [17]).

On the other hand, the length of the gradient used may not have included the extremes. We, however, argue that this may not be the case, as the most arid site is four times drier than the least arid one, involving great differences in water availability. Instead, a different interpretation of the cubic relationship might be related to biogeography. Given the big study area, the ups and downs in the curve might be related to changes in the flora given the wide latitudinal span of the study area. One way to verify this is by assessing the patterns in a more restricted portion of the region, one having a more similar flora. The region between latitudes 20 and 24°S falls in this category; nevertheless, a similar cubic relation is obtained here. In theory, this should be expected, as the theory of interactions along stress gradients does not assume necessarily a uniform flora. Moreover, if we are faced with extreme gradients, we are expected to find floristic changes along those gradients, as different taxa should be adapted to the different portions of the gradient. Therefore, the pattern may not be related to floristics, although this interesting possibility should be further evaluated through a study that addresses this specific question.

One important aspect that is not considered by co-occurrence indexes is related to the fact that they do not measure intraspecific associations. Hence, if there had been intraspecific facilitation at our study sites, our analysis could not have captured the pattern it generated.

Alternatively, both gradient hypotheses, the one that predicts stronger facilitation with aridity (SGH, [6]) and the one which postulates a facilitation peak at intermediate stress levels (the hump-shaped relationship [11], [12], [13]), may not hold in the Andean gradient for these life forms. In this sense, our regression analysis for all species supports the explanation put forward recently by Soliveres et al. [17], which do not predict major changes in the importance or intensity of facilitation along environmental gradients because, at any given point along the gradients, there will always be species experiencing stress. A given stress does not affect all species of a community equally, but mainly those that are at their distributional limits [14], [15], [17], and the proportion of these species should be essentially similar from point to point across the gradient. One way or another, our community-level analysis shows that the stress gradient hypotheses do not hold in the subtropical arid Andes, or at least our results show that any pattern consistent with these hypotheses is weak at a community-scale, as the response of the dominant life forms is governed by idiosyncratic factors of each community over the effects of aridity. This implies that the importance of facilitation (*sensu* Brooker et al. 2005 [52]) does not increase with aridity in these mountain systems.

In synthesis, facilitation appears to be a structuring process only for woody and cactus species in hot deserts of the subtropical Andes. There is, however, no monotonic increase in positive plant associations with aridity. Our data support the hypothesis that facilitation does not change linearly with aridity, or at least show that other factors are more important for the community than a response to reduced water availability. In any case, the association patterns found along the aridity gradient of this region provide no support for the SGH.

## Supporting Information

Table S1 Data matrices for the all species case.
(XLS)

Table S2 Data matrices for the herb species case.
(XLS)

Table S3 Data matrices for the shrub/cactus species case.
(XLS)

Table S4 Data matrices for the shrub species only case.
(XLS)
